# “We’re tolerant and they’re prejudiced”: Same-sex marriage supporters’ and opponents’ perceptions of supportive and oppositional claims

**DOI:** 10.1371/journal.pone.0286063

**Published:** 2023-08-29

**Authors:** Michael J. Platow, Clinton G. Knight, Dirk Van Rooy, Martha Augoustinos, Daniel Bar-Tal, Russell Spears

**Affiliations:** 1 School of Medicine and Psychology, The Australian National University, Canberra, Australian Capital Territory, Australia; 2 College of Healthcare Sciences, James Cook University, Townsville, Queensland, Australia; 3 Faculty of Design Sciences, University of Antwerp, Antwerp, Belgium; 4 School of Psychology, The University of Adelaide, Adelaide, South Australia, Australia; 5 School of Education, Tel Aviv University, Tel Aviv, Israel; 6 Faculty of Behavioural and Social Sciences, University of Groningen, Groningen, The Netherlands; Universite Paris Pantheon-Assas, FRANCE

## Abstract

The current research examined the proposition that debates over same-sex marriage are characterized, at least in part, by conflicting understandings about what is and is not prejudiced, normative and true. Toward this end, Australians’ (N = 415) prejudice judgements of supportive and oppositional statements toward same-sex marriage were measured and analysed with analyses of variance. Unsurprisingly, same-sex marriage supporters perceived a supportive statement as unprejudiced, tolerant, truthful, in pursuit of individual liberty, and normative; oppositional statements were seen in precisely the opposite manner. Same-sex marriage opponents, however, disagreed, instead judging an oppositional statement as unprejudiced, tolerant, truthful, in pursuit of individual liberty, and normative; it was a supportive statement that was seen as relatively prejudiced. These effects remained even after controlling for independent expressions of in-group favouritism. The current data align with a collective naïve realism perspective, in which group members see their own views as veridical and those of disagreeing others as biased. We argue that prejudice-reduction efforts must be instantiated to facilitate a common in-group identity between supporters and opponents to enable consensus over facts and, ultimately, what is and is not prejudice. Without this consensus, each side of the political debate may simply hurl the pejorative label of “prejudice” against the other, with likely little opportunity for social influence and social change.

## Introduction

Opposition to same-sex marriage reflects the denial of opportunities to one group of people that are routinely afforded to another. For this reason, it is understood by many as inherently unjust and prejudiced [e.g., 1–3]. Empirically, research confirms this understanding, with positive associations observed between same-sex marriage opposition and both direct [4] and indirect [e.g., 5, 6] measures of prejudice. In the current paper, however, opposition to same-sex marriage, as well as support for it, are examined through a different conceptual and empirical lens. In particular, we do not assume from the outset that one group of people in this debate (e.g., supporters) is somehow rational and just, while the other (e.g., opponents) has a flawed psychology and a flawed sense of justice. Instead, we propose that political debate over same-sex marriage is characterized, at least in part, by conflicting understandings about *what constitutes prejudice itself*. It is this intergroup conflict over subjective understandings of what is and is not prejudice that forms the empirical basis of the current paper.

Below we present a brief review of social-psychological conceptualizations of prejudice, with a particular focus on the variability of these conceptualizations and their ultimate bases in social norms. From this, hypotheses are derived that are evaluated in an experiment measuring perceptions of prejudice, tolerance and truth in the context of the 2017 Australian national poll on support for, or opposition to, same-sex marriage [7].

### Psychological conceptualizations of prejudice

Social psychology has a long history examining prejudice [e.g., 8–11]. Most of this work has focused on the expression of negative intergroup attitudes, and has been highly successful in identifying the causes of these attitudes as well as various means of changing them [e.g., 12–15]. Recent analyses, however, have highlighted challenges inherent in the prejudice concept that make it a variable and changing target in terms of both theoretical understanding and prejudice-reduction efforts [16, 17].

For example, although negative intergroup attitudes characterize most understandings of prejudice [16], not all negative intergroup attitudes are equally recognized as such. In fact, some negative intergroup attitudes are, at times, seen as just and accurate descriptions of reality (e.g., negative attitudes toward child molesters; [17, 18]). At the same time, not all prejudice research has focused exclusively on the expression of negative attitudes. Indeed, Allport’s [8] original analysis recognized that positive attitudes can also be understood as prejudiced (see also [19]). This variability in attitude valence is exposed in the tensions inherent in the identification and expression of “modern,” “symbolic,” and “benevolent” prejudice [20–22]. In contrast to more explicitly negative “traditional” or hostile forms of prejudice, modern/symbolic/benevolent prejudiced attitudes are often congruent with other broadly accepted group norms and values (e.g., hard work, religious values). Unfortunately, this variability in understanding means that psychologists often end up examining not a single concept, but multiple ones, often with different underlying assumptions about the social and psychological processes underlying them [23].

What consensus there seems to be among both researchers and lay people alike is that prejudice is about groups and it is bad [e.g., 24]. Effectively, prejudice is understood as the expression of wrong or inappropriate attitudes about groups and group members [25, 26]. Even conceptualizing prejudice as a form of pre-judgement, for example, assumes a more appropriate and correct form of judgement. This “enlightenment” perspective, thus, assumes that prejudice emerges because people do not have access to the full understanding of the true nature of groups and group members [27]. This is demonstrated in work that links lower cognitive abilities to prejudice [e.g., 28], implicitly suggesting that higher cognitive abilities would allow one to gain a more accurate understanding of the “facts.” Similarly, an explicit purpose of intergroup contact to reduce prejudice is that of “learning about the outgroup” [29, p. 65]. Again, this perspective presupposes that prejudice is the consequence of inaccurate beliefs. And, of course, educational programs that directly attempt to teach non-prejudiced ways of understanding [30] once again presuppose that prejudice emerges from inaccurate beliefs.

Another way of characterizing this perspective is by recognizing that it assumes prejudice emerges when attitudes and beliefs diverge from normative standards of accuracy and correctness [17]. Critically, however, such normative standards have their own bases in the social psychology of group life [e.g., 31, 32]. Indeed, social psychologists have long acknowledged that divergent understandings of the same reality can be (and often are) determined by people’s group memberships and the nature of the intergroup relations [e.g., 33–35]. In the context of the current analysis, when group members do not agree upon what the facts are, or how to determine facts in the first place, then they are not likely to agree on what attitudes are or are not prejudiced as well [36]. This is seen very clearly in political debates over same-sex marriage [37, 38]. Each side of the debate harnesses facts to support its position, and neither considers itself to be prejudiced (a process that extends to prejudice debates beyond the same-sex-marriage; [18, 39]).

When admission of prejudice is at least hinted at, it is often done with the disclaimer that “I’m not prejudiced, but…” [40, 41]. The use of such disclaimers reveals speakers’ awareness that their attitudes may be *perceived* as prejudiced, and that work is needed to avoid incurring the prejudice label [42]. The need for this work emerges because the prejudice label itself is seen as pejorative by implying irrationality and non-normative behaviour [43, 44]. Indeed, being simply labelled as prejudiced has the potential to evoke feelings of offense and anger [45], so that people actively seek to avoid this label across a variety of intergroup contexts [e.g., 46, 47]. Opponents to same-sex marriage, for example, are actually “sensitive to the possibility of being labelled homophobic” [38, p. 11].

### The current research

Currently, we propose that the identification of attitudes as prejudiced or not is always, and necessarily, done with reference to perceived norms and values of one’s salient group membership, situated within a particular intergroup context [e.g., 17, 48]. In this way, people will view attitudes they share with a salient in-group to be rational, veridical and unprejudiced. In contrast, non-normative attitudes will be seen as irrational, untrue and, in the case of intergroup attitudes, prejudiced. Note that the absolute valence of the attitudes is irrelevant in this analysis, as are independent assessments of veracity. Of course, as scientists, we do not deny an independently verifiable reality. However, our assumption is that the means by which people confirm this reality is tied to their salient group memberships (just as ours is tied to science). In this manner, different people’s understandings of truth and prejudice will be tied to their group memberships. To the degree that there is variability in group memberships, then there will also be variability in understandings of prejudice, as noted above.

In the case of attitudes toward same-sex marriage, this leads us to predict that (H1a): Supporters of same-sex marriage will judge expression of support for same-sex marriage as unprejudiced and tolerant, and opposition as prejudiced and intolerant. In some ways, H1a is unremarkable, as it is congruent with our opening observation. However, we also predict that (H1b): Opponents of same-sex marriage will judge expression of opposition to same-sex marriage as unprejudiced and tolerant, and support as prejudiced and intolerant. Moreover, we predict that (H2): Members of each (supporting or opposing) opinion-based group will see their own views as both veridical and normative of the broader society, while the other group’s views will be seen as false and non-normative. We tested these hypotheses in a sample of Australians and, as such, this “broader society” was operationalized as Australians.

## Method

### Participants and design

Participants were sampled using Qualtrics Australia panel service. Data supplied by this service included an initial sample of 460 participants who satisfied several inclusionary criteria, including: (1) correctly responding to an experimental manipulation check (described below), (2) correctly responding to all three included conscientious responder items [49], and (3) indicating that they had well-informed views about the same-sex marriage debate. Moreover, a relatively balanced male-female gender ratio was sought, as was a basic age stratification. Of these, 44 participants asked that their data not be included in the final analyses (an ethical requirement imposed upon the current study). Finally, one participant under the age of 18 years was excluded from analyses (an ethical requirement and a requirement of the study design, as the minimum Australian voting age is 18 years).

Participants were sampled on 5 November, 2017–8 November, 2017. These were the final days during which the Australian government held a non-binding, national Postal Survey of all eligible voters’ support for, or opposition toward, same-sex marriage. Postal Surveys were distributed to Australians from 12 September, 2017 and were required to be received by the government no later than 7 November, 2017. As such, at the time the current study was conducted, considerable public debate about the Postal Survey had already occurred [e.g., 50, 51]. Participation rate in the Postal Survey was 79.5% of eligible voters (all Australians over the age of 18 are required by law to register to vote; [7]).

Of the final sample, 199 identified as male and 216 identified as female. Ages ranged from 18–89 years (median = 50). Two-hundred, twenty participants identified as “White”/“Caucasian”/“Anglo” or simply “Australian;” of course, someone who identifies as “Australian” could also be of, for example, Aboriginal, Asian or African descent, making it difficult to present accurate frequencies of the remainder of the ethnic composition. One-hundred, sixty-three participants had completed some level of university education, while 126 completed tertiary vocational education or apprenticeships. Three-hundred, seventy-four participants indicated that English was their first language.

Of particular relevance to the current research on same-sex marriage, a total of 254 people were or had been married (189 people reported being currently married, 10 separated but not divorced, 43 divorced, and 12 widowed); a further 48 people reported being in de-facto relationships. In terms of sexual orientation, 335 people indicated that they were “only attracted to members of the opposite sex;” twenty-seven indicated being “only attracted to members of the same sex,” 32 people indicated being attracted to members of the same sex, opposite sex and transgender persons, 10 indicated not “really that attracted to other people,” six indicated that their “preferences are more complicated than the options provided,” and five preferred not to say. Finally, 252 participants (60.7% of the sample) identified as supporters of same-sex marriage while 163 participants (39.3% of the sample) identified as opponents of same-sex marriage. These numbers compare quite well with the final distribution of supporters (61.6%) and opponents (38.4%) in the broader population based on the Postal Survey.

Each participant was randomly assigned to one of two between-participants conditions in which they were presented with a statement either supporting or opposing same-sex marriage (described below). The research design was, thus, a 2 (measured participant support for or opposition toward same-sex marriage) x 2 (manipulated stimulus statement supporting or opposing same-sex marriage) between-participants factorial.

### Materials and procedure

Participants first read a Statement of Informed Consent. Upon agreeing to continue, they read a brief introduction reminding them about the same-sex marriage Postal Survey, stating “…there are a lot of different views on the matter. In the current survey, we’re interested in what average Australians think about this matter and what they think about some of the opinions people have been expressing.” This was followed by a statement informing participants that they would be presented with “…one randomly selected opinion on the matter. Please read the opinion and consider what the author has said. We will then ask you about your own opinions about what the author said.” The subsequent screen then informed participants that “a statement is now being randomly chosen,” which was followed by one of two statements representing the experimental manipulations. Each statement began with “Marriage has been around for centuries. What makes a marriage though is a life-long romantic commitment between….” The statement in support of same-sex marriage continued with “….two people, whoever they may be. That is why I’m voting YES in the survey.” In contrast, the statement in opposition to same-sex marriage continued with *“*…a man and a woman, whoever they may be. That is why I’m voting NO in the survey.”

These two stimulus statements were constructed to be relatively pallid in nature, simply reaffirming support for, or opposition toward, same-sex marriage. We intentionally did *not* provide explicitly negative statements. Indeed, even the oppositional statement was affirmative about a particular understanding about human relationships. Moreover, the statement in support of same-sex marriage is, at least on the surface, explicitly and intentionally devoid of attributes that would allow it to be categorized as prejudice by most conceptual accounts (e.g., negative intergroup attitudes or feelings; [16, 17]). Both statements also include an attribute that, with deeper reflection, participants could have easily recognized as a falsehood: that there is a centuries’ long tradition of marriage involving life-long romantic commitments. This explicitly ignores, for example, arranged and forced marriages in which romance and commitment may play little to no part. Our goal in constructing the stimulus statements in this nature was *not* simply to reaffirm negative intergroup statements as prejudice. Instead, our goal was to identify conditions under which statements that would not otherwise be formally categorized as prejudice would be judged as such by lay perceivers. Despite our pursuit of experimental control in the design of our stimuli, we recognize that the public discourse on this issue at the time of the national survey was often more emotionally charged and even hostile than our more staid manipulations; participants may well have encountered this hostility outside the context of this study, either directly or indirectly (e.g., traditional media, social media). We reflect on this briefly in our Discussion.

A single multiple-choice manipulation check question followed the presentation of the statement, stating, “Just to make sure you understand a bit about the statement…the author of the above statement is going to vote….” Response options available to participants were simply “yes”, “no”, and “did not say.” Only data from participants who correctly answered this question were provided by Qualtrics panel service. To encourage participants to reflect on the statement, they were then given a free-response opportunity to write their thoughts in response to the statement, “My own views about the statement above are…” (the data measured here will form the basis of a separate publication).

At this point, the primary dependent variables were presented. Participants were asked to “use the rating scales below to record your thoughts about the statement… ‘I think the statement is… .’” A series of 40 items comprising six conceptual scales were randomly presented to each participant. The items, the scales they comprised, and Cronbach’s alpha for each scale are presented in Table 1. As can be seen, the scales measured perceptions of prejudice, tolerance, truth, Australianness (i.e., normativeness within the broader society), individual liberty, and sinfulness. The individual liberty and sinfulness scales were not related to our hypotheses, and were included for exploratory purposes. Individual liberty was included because arguments in favour of same-sex marriage often rely on this concept. Similarly, arguments against same-sex marriage often rely on the concept of sinfulness. Although all of the scales were uniquely developed for this study, similar methods have been used in other research [e.g., 48, 52]. All responses were measured on seven-point Likert scales (1 = strongly disagree, 7 = strongly agree).

**Table 1:** Evaluative scales and individual items (and Cronbach’s alphas/Pearson correlation) presented to participants to evaluate the stimulus statements.

Prejudice	Tolerance	Truth	Australianness (i.e., normativeness)	Individual Liberty	Sinfulness
α = .95	α = .94	α = .96	α = .94	α = .84	*r* = .32**
prejudice	Tolerant	truth	consistent with Australian values	individual rights	sinful
stereotyping	Inclusive	correct	just the Australian way	individual expression	traditional*
discriminatory	Accepting	factual	what Australia should be striving for	freedom of speech	
intolerant	Welcoming	objective	typical of what most Australians believe	freedom of religion	
preconceived	Progressive	logical	“fair dinkum”***	democratic	
biased	Multicultural	reasonable	unAustralian*	independent minded	
unjustified	unaccepting2*	legitimate			
unfair		valid			
misguided		unrealistic*			
offensive					

*Reverse coded

**Pearson correlation is reported because Cronbach’s alpha is inappropriate for two items, *p* < .001.

***This is an Australian colloquial expression used to confirm the truth or validity of a statement. We included it in the Australianness scale rather than the truth scale because of its clear cultural association with being Australian.

Participants were then presented with a new set of 12 randomly-presented evaluative items [e.g., 53] and were asked to provide judgements of “yes voters” and “no voters” (counter-balanced) in general (i.e., not of the stimulus statement, as above). The items were “aggressive” (reverse coded), “competent,” “friendly,” “honest,” “intelligent,” “intolerant” (reverse coded), “pleasant,” “sincere,” “skilful,” “threatening” (reverse coded), “trustworthy,” and “warm.” All responses were measured on a six-point scale (1 = not at all; 6 = extremely). These items allowed us to measure directly in-group favouritism in the current intergroup context.

Finally, participants answered additional questions, including demographics. The additional questions included scales measuring social dominance orientation, right-wing authoritarianism, religiosity, and endorsement of political correctness attitudes; these will form the basis of a separate paper. Including these four scales as covariates did not alter the pattern of currently reported results. In the demographic section, participants were presented with a single item measuring the extent of their social identification with their opinion-based group, “I identify with YES [NO] voters” (1 = fully disagree, 7 = fully agree; [54]). The experiment ended with a complete written debriefing, including an explanation of the fictitious nature of the attitude statement presented.

## Results

A single missing value was identified and replaced with an estimate derived from the expectation maximization procedure.

In an attempt to control for personal self-interest, each of the following analyses was re-conducted with a reduced sample excluding participants who could potentially benefit personally from the institution of a same-sex marriage law (i.e., participants who expressed same-sex attraction). The pattern of results in all cases was identical to what we currently present.

### Social identification

A 2 (measured participant support for or opposition toward same-sex marriage) x 2 (manipulated stimulus statement supporting or opposing same-sex marriage) between participants analysis of variance (ANOVA) on participants’ levels of social identification with their in-group revealed no statistically significant main or interaction effects. Importantly, there was no significant difference in levels of social identification between supporters (*M* = 6.43, SEM = .08) and opponents (*M* = 6.21, SEM = .10), *F*(1,411) = 2.93, *p* = .09. Participants’ overall levels of social identification with their supporting or opposing in-group was extremely high (*M* = 6.34, SEM = .06). A full 85.32% of supporters and 80.98% of opponents reported social identification levels of either 6 or 7 on the seven-point scale. Because of the highly skewed nature of this measure, we did not employ it further as a potential moderator in the primary analyses.

### Primary analyses

Independent 2 x 2 ANOVAs were conducted on each of the six evaluative scales described in Table 1. Statistically significant main effects for the experimental condition occurred on perceptions of prejudice [*F*(1,411) = 56.35, *p* < .001, η^2^_partial_ = .12], tolerance [*F*(1,411) = 158.97, *p* < .001, η^2^_partial_ = .25], and sinfulness [*F*(1,411) = 163.66, *p* < .001, η^2^_partial_ = .29]. Participants overall perceived the “yes” statement to be less prejudiced (*M* = 3.15, SEM = .09), more tolerant (*M* = 4.65, SEM = .08) and more sinful (*M* = 4.21, SEM = .09) than the “no” statement (*M*_*prejudice*_ = 4.01, SEM = .08; *M*_*tolerant*_ = 3.38, SEM = .08; *M*_*sinful*_ = 2.65, SEM = .08). Statistically significant main effects were also observed for participants’ own support or opposition on perceptions of prejudice [*F*(1,411) = 11.26, *p* = 003, η^2^_partial_ = .02] and sinfulness [*F*(1,411) = 41.36, *p* < .001, η^2^_partial_ = .06]. Same-sex marriage supporters perceived the statements overall to be more prejudiced (*M* = 3.75, SEM = .07) and less sinful (*M* = 3.11, SEM = .08) than same-sex marriage opponents (*M*_*prejudice*_ = 3.41, SEM = .09; *M*_*sinful*_ = 3.76, SEM = .10).

**Fig 1:**
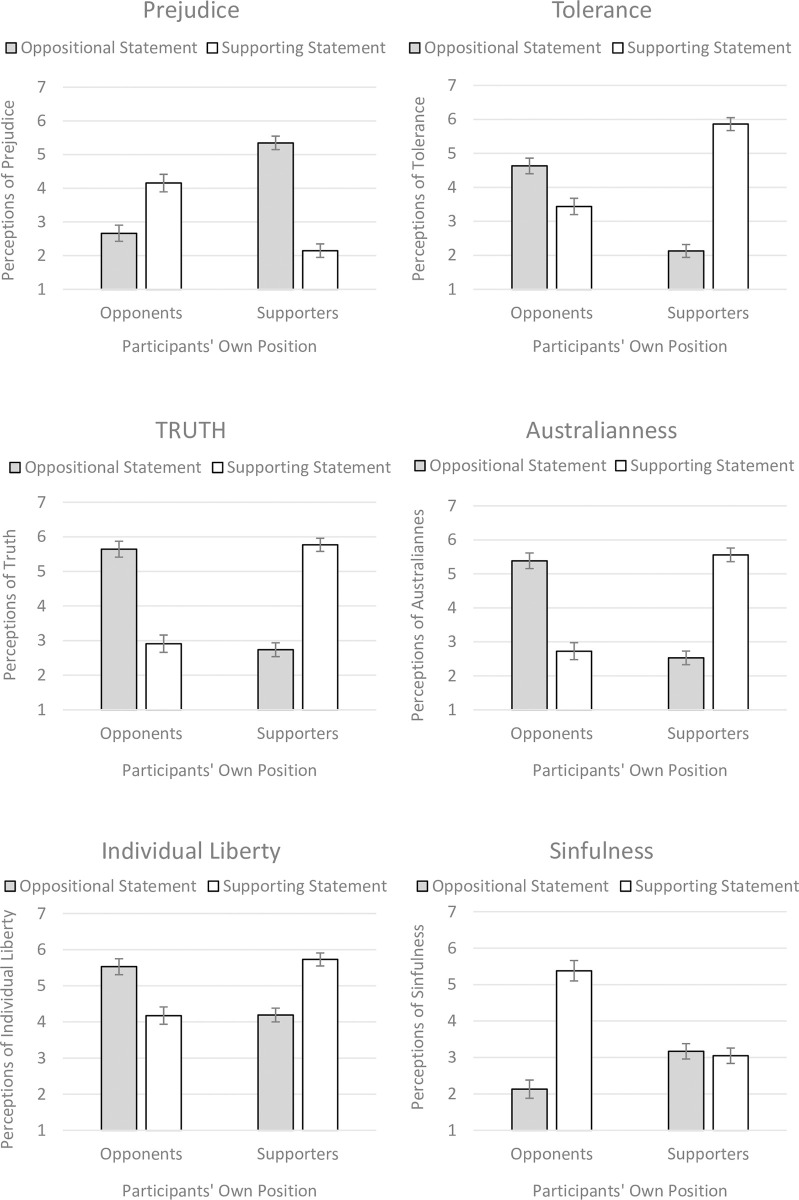
Statistically significant interactions between measured participant support for/opposition toward same-sex marriage and manipulated stimulus statement supporting or opposing same-sex marriage. The bars represent 95% confidence intervals.

More relevant to the hypotheses, participants’ responses on each of the six dependent variables entered into highly significant disordinal interactions. The *F*-statistics are presented in Table 2, and the interactions are presented in each of the panels of Fig 1. Consistent with our hypotheses, both supporters and opponents of same-sex marriage judged the in-group statement as being tolerant, truthful, and Australian, while the out-group statement was clearly perceived as relatively prejudiced. A similar pattern was also found in judgements of individual liberty. The only interaction that deviated from this symmetrical pattern was participants’ judgements of sinfulness. Along this dimension, only same-sex marriage opponents perceived a difference between the statements, with a supporting statement being perceived as more sinful than an opposing statement. Simple effects comparisons within the supporter and opponent groups were all statistically significant (all *p*s < .001) except for supporters’ judgements of sinfulness (*p* = .39).

**Table 2:** F-statistics, significance levels, and η2partial for the interaction between measured participant support for/opposition toward same-sex marriage and manipulated stimulus statement supporting or opposing same-sex marriage.

Scale	*F*(1,411)	*p*	η^2^_partial_
Prejudice	424.04	< .001	.51
Tolerance	597.42	< .001	.56
Truth	660.37	< .001	.62
Australianness	637.00	< .001	.61
Individual Liberty	183.48	< .001	.31
Sinfulness	189.91	< .001	.32

The common pattern replicated across the panels of Fig 1 suggests that the six scales are likely to be correlated. Separate analyses confirms this (see Table 3). However, we chose to maintain the independent ANOVAs (rather than averaging across all items) precisely to reveal this common pattern emerging across conceptually different judgements. Concerns of enhanced Type 1 errors are mitigated against in light of the currently obtained high levels of statistical significance. We also chose to maintain our original conceptual scales rather than empirically deriving them because a separate principal-components analysis with varimax rotation yielded a solution in which multiple items loaded highly across several components. As such, the solution was suboptimal in identifying independent scales despite the varimax rotation.

**Table 3:** Pearson correlations between each of the six evaluative scales (all *p*s < .001).

	Evaluative Scales				
	Prejudice	Tolerance	Truth	Australianness	Individual Liberty
Tolerance	-.86				
Truth	-.84	.87			
Australianness	-.82	.86	.93		
Individual Liberty	-.62	.71	.75	.72	
Sinfulness	.33	-.31	-.53	-.52	-.44

Although these common patterns exist (with strong disordinal interactions), it is also visually apparent that the magnitude of the pair-wise difference in ratings within same-sex marriage supporters is often different from that within same-sex marriage opponents. To examine these apparent differences, we recoded our data to allow us to conduct 2 (measured participant support for or opposition toward same-sex marriage) x 2 (judgement of an *in-group* statement or judgement of an *out-group* statement) between participants ANOVAs. In these new analyses, the interactions reported above will manifest as simply main effects for the judgement of in-group vs. out-group statements. However, a significant interaction will now indicate that the magnitude of this main effect differs between supporters and opponents. These significant interactions were observed for three judgements only: prejudice [*F*(1,411) = 56.35, *p* < .001, η^2^_partial_ = .12], tolerance [*F*(1,411) = 137.26, *p* < .001, η^2^_partial_ = .25], and sinful [*F*(1,411) = 163.66, *p* < .001, η^2^_partial_ = .29]. We consider the implications of these differences in our Discussion.

### In-group favouritism

The 12 evaluative items used to measure in-group favouritism had high internal reliability for judgements made of same-sex marriage supporters (α = .96) and same-sex marriage opponents (α = .96). As such, means of the two sets of 12 items were calculated for each participant and analysed in a 2 x 2 x 2 mixed ANOVA, with the final factor being the within-participants judgements of each of the two groups. The only statistically significant effect to emerge was the two-way interaction between participants’ own group and the group being evaluated, *F*(1,411) = 389.65, *p* < .001, η^2^_partial_ = .49. Strong in-group favouritism was found: Supporters of same-sex marriage judged fellow supporters more favourably (*M* = 4.77, SEM = .07, 95% CI[4.64, 4.90]) than opponents (*M* = 3.03, SEM = .07, 95% CI[2.90, 3.17]), while opponents of same-sex marriage judged fellow opponents more favourably (*M* = 4.69, SEM = .09, 95% CI[4.52, 4.86]) than supporters (*M* = 3.20, SEM = .08, 95% CI[3.04, 3.36]).

### Post-hoc analyses: Controlling for in-group favouritism

We recognize that the observed interactions from the primary analyses presented in Fig 1 are similar to the in-group favouritism interaction also observed. It may, thus, be that the Fig 1 patterns are simply another reflection of generic in-group favouritism. To test this, we calculated the difference in in-group and out-group evaluations for each participant. Each of the primary ANOVAs was re-run, this time treating the in-group favouritism measure as a covariate. All interactions remained significant (all *p*s < .001) and the patterns remained unchanged. Although there may be a component of in-group favouritism contributing to the Fig 1 patterns, these effects are certainly not isomorphic with it. The effects in Fig 1 are not accounted for merely by in-group favouritism.

### Post-hoc analysis: Examining mediation

In our Introduction, we reasoned that group members are not likely to agree on what is and is not prejudice if they do not agree on what the facts are in the first instance. We explored this possibility by examining whether perceived truth mediated the relationship between the interaction of support/opposition toward same-sex marriage and the supporting/opposing stimulus statement on judgements of prejudice (including the measure of in-group favouritism as a covariate). Using Hayes’ Process model, the index of moderated mediation was significant (IMM = -3.86; 95% CI[-4.45, -3.29]). The interaction process that we observed in the prediction of prejudice judgements is, indeed, mediated by judgements of truth.

### General discussion

The current research examined group members’ perceptions of what is and is not prejudice in the context of a political debate over legalizing same-sex marriage in Australia. Rather than assuming from the outset that one side of the debate was inherently just and correct while the other was inherently prejudiced, we asked the group members themselves to provide their own judgements of claims supporting or opposing same-sex marriage. Supporters of same-sex marriage saw opponents’ views as prejudiced, intolerant and untruthful, while their own views where perceived as unprejudiced, tolerant and truthful. As noted, this finding is broadly unsurprising, as opposition to same-sex marriage denies a group of people opportunities that others are granted. What is more novel is that opponents of same-sex marriage did not share this view. In fact, they saw their own views, too, as unprejudiced, tolerant and truthful, while supporters’ views were perceived as relatively prejudiced, intolerant and untruthful. Although these judgements have an element of in-group favouritism, the patterns remained even after statistically controlling for direct expressions of in-group favouritism.

We propose that these are genuine views held by members of each group. The patterns were not simply about hurling the pejorative prejudice label against out-group members, but about grounding the in-group perspective in subjective understandings of truth and in-group normativeness. Group members on each side of the debate were essentially saying, “*We* see reality as it truly is, and our fellow Australians concur with us. *They* do not understand this reality and are, hence, relatively prejudiced and intolerant.” This pattern has important implications for the enlightenment perspective of prejudice described in the Introduction. This perspective assumes that there are objectively prejudiced people and objectively enlightened people, and that enlightened people must educate the prejudiced if there is to be appropriate social change. The current data suggest, however, that all people see themselves as enlightened (at least on average). To this degree, each side may feel no need to receive further education and, moreover, may feel responsible to “educate” those on the other side. In this case, such education becomes a social influence attempt [55] to persuade out-group members to see the world as they (the educators) do themselves.

These data thus pose clear conceptual and applied challenges. On the one hand, they compel researchers and social-change agents alike to reflect more deeply on the concept of prejudice itself, and articulate precisely the parameters around which the concept is understood and defined. As we observed, recent analyses have highlighted variable disciplinary definitions of prejudice that often invoke variable social and psychological processes. This, in itself, means that researchers’ failures to come to a conclusive understanding of prejudice emerges from researchers’ failures to come to a definitional consensus. The current data, however, clearly demonstrate that this lack of consensus is paralleled within the broader community. It is here where the heterogeneity of understandings poses a challenge to social change efforts. For example, same-sex marriage opponents are likely to be immune to (at best), if not actively push back against (at worst), efforts to change their attitudes, particularly if their own attitudes incurred the label of “prejudice” in the process. This would be, based on the current data, because opponents again see their own views as veridical, normative and tolerant.

The perspective of same-sex marriage opponents warrants further reflection. The opening sentence of this paper identified same-sex marriage opposition as the denial of opportunities to some that are afforded to others. By this very description, then, how can opposition ever be seen as anything other than prejudice, even by opponents? In answering this, it may suit supporters to maintain claims that opponents are misinformed, uneducated and simply cognitively lazy. But the current data suggest other possible explanations. Same-sex marriage opponents see their own views as being veridical and in accord with individual liberties, while supporters’ views are seen as sinful and, in fact, relatively prejudiced. Although the current research did not include independent measures of cognitive abilities, opponents do appear to be constructing a rational argument for their views *as they understand them*. Moreover, while supporters may see opponents as prejudiced against people seeking same-sex marriages, opponents may well see supporters as prejudiced against themselves (i.e., opponents). Indeed, previous research has clearly articulated how opponents frame their views within particular normative values (as we have currently argued; e.g., [56]). The legalization of same-sex marriage, then, may be perceived by opponents as denying *them* opportunities (e.g., individual liberty and freedom of religion) routinely afforded others (and, hence, is prejudiced).

We do note, of course, that when expressing judgements of prejudice in particular, opponents’ mean views of a supporting statement were around the midpoint of the current response scale. As such, it is not so much that supporting statements are seen as prejudiced, *per se*, but simply *more* prejudiced (or less unprejudiced) that opposing statements. In this manner, opponents’ judgements may still be tempered, at least in part, by additional social norms within Australia regarding the nature of justice and fairness (the same applies for their judgements of tolerance). As previous authors have noted [57, 58], justice rules are bounded within social categories. Regardless of opponents’ views of the relative Australianness of the statements, when providing their judgements of prejudice (and tolerance), the broader self-categorization as Australian may have been salient, thereby leading them to include supporters as well as opponents in the same moral community to which the justice rules applied. Clearly, this potential process is ripe for more detailed empirical examination.

Ultimately, the current conceptual and empirical work highlights an alternative path to prejudice reduction to those that have routinely been employed by psychologists and social change agents. We propose that debates over prejudice are, at least in part, debates over truth. But rather than assuming *a priori* that one side has privileged access to this truth while the other does not, we should enter into the debate with the recognition those on each side share the same psychological *processes* despite not sharing the same attitudes.

Here Ross and Ward’s [59] analysis of naïve realism becomes particularly useful. These authors propose that people believe that they, themselves, “see entities and events as they are in objective reality, and that [their] social attitudes, beliefs, preferences, priorities…follow from a relatively dispassionate, unbiased…apprehension of the information…” (p. 110). Ross and Ward also propose that people believe other “rational social perceivers” (e.g., fellow in-group members) will see the world similarly, while those who do not are “lazy,” “irrational,” or “biased” (p. 111). The currently obtained patterns of data could not be more closely aligned with Ross and Ward’s analysis. The primary difference is that Ross and Ward’s analysis focuses more on idiosyncratic perceptions aligned with personal identities (i.e., “*I* see things as they are in reality.”). Our work expands this to shared perceptions aligned with social identities (i.e., “*We* see things as they are in reality.”), just as has been shown in independent research related to the Israeli-Palestinian conflict [60].

The post-hoc analysis of our data provide preliminary confirmation of the role that truth perceptions are likely to play in mediating the relationship between participants’ own opinion-based group memberships (same-sex marriage support or opposition) and their prejudice judgements of supportive or oppositional statements. Clearly, when people fail to agree on what is and is not true, they are not likely to agree on what is and is not prejudice. An important conceptual and applied consideration of the collective naïve realism currently observed is that prejudice reduction efforts ought to focus not so much on the truth *per se* (which each side believe they have), but on the *means* by which the truth is determined. In this way, debates over prejudice can be reframed as debates over epistemologies. Once intergroup consensus and shared norms are obtained over how truth is determined, then consensus over what is and is not prejudice should follow.

None of this is trivial in the context of same-sex marriage debates. As of writing this paper, same-sex marriage remains legally unavailable to people in 85% of the world’s countries [61]. Millions of people are denied this simple but valued social relationship (a relationship that often brings with it other economic and social benefits). Efforts to change this state of affairs will be stifled if opponents are cast as irrational and uneducated. We can see this problem in other political domains, such as in the support for populist ideologies [62] and anti-vaccine attitudes [63]. Consensus needs first to be built over what the relevant facts are and how they are determined. This is no simple matter, as each side may turn to ideologically incompatible epistemologies (e.g., science and religion). But it is precisely here where the prejudice-reduction practices that social psychologists have developed become relevant and useful. The critical point, however, is to employ these *not* between, say, heterosexual and homosexual people, nor between religious and non-religious people, nor between (supposedly) educated and uneducated, and not even between prejudiced and non-prejudiced people. These practices need to be employed simply between supporters and opponents of same-sex marriage. Moreover, we suggest that it is not sufficient simply to build intergroup friendships to reduce or eliminate in-group favouritism. Instead, the goal must be to develop a common shared social identity [14, 64] that will form the basis for the requisite shared epistemology. Again, this is no simple task, as we are saying nothing less than prejudice reduction is needed. But the current research does provide guidance by proposing a mechanism through which we can pursue social change.

Before we conclude, it may be worth reflecting on the broader context in which this work was conducted. As we noted above, the debate in Australia became, at times, emotionally charged and hostile. Members of the broader Australian LGBTIQ community, along with their parents, reported increased experiences of negative attitudes and behaviours directed toward them, leading them to feel anxious, fearful and depressed [65–67]. This broader negative discourse, of course, may have impacted upon how our participants understood the claims we presented. Indeed, it may well have contributed to the larger difference in judgements among the “yes” supporters than the “no” supporters. At the same time, we can not deny the strength of the disordinal interactions we observed; our effects were not driven solely by the judgements of “yes” supporters. The current findings thus align with other recent analyses on the partisan nature of truth–or, at least, subjective understandings of truth–that have been observed in the absence of intergroup hostility, among experimentally-created, randomly assigned groups [68].

### Limitations and future research

Despite clear strengths of the current research (e.g., a broad cross-section of the Australian population, sampling during a time when people were likely to have well-formed attitudes), we recognize that this work is not without limitations. First, by its very nature, our use of opinion-based groups means that relationships we observe are necessarily correlational. We have argued, for example, that people base their judgements of prejudice upon their understandings of truth. This assumes a causal order of self-categorization (into one group or another) that leads to truth determination that leads to prejudice determination. Clearly, there are other possible causal orderings. The most obvious alternative causal ordering is that truth and prejudice determinations are simply developed *post facto* to justify one’s *a priori* negative intergroup attitudes in a motivated reasoning manner [69]. Indeed, we believe that, in some cases, this may well be the operative process. But the fact that directional motivated reasoning may guide some people’s judgements does not negate the possibility of collective naïve realism as we suggested above. Separation of these processes will require experimental research, not simply surveys.

Second, we recognize that our conceptual analysis has cast opponents of same-sex marriage in potentially a more favourable light than many readers would like. In this way, we may be seen as nothing more than apologists for prejudice (much as psychologists have been in other domains, as in the justification of prejudice against Aboriginal Australians; [70]). We acknowledge this, but disagree. Instead, as researchers, we are seeking to understand social and psychological processes that underlie what otherwise are labelled as prejudiced attitudes. Moreover, the current analysis seeks to understand how and why the same-sex marriage debate remains entrenched. If we simply cast dispersions against those with attitudes we do not share, we will, of course, be open ourselves to accusations of prejudice. Currently, we are, through the epistemology of science, attempting to understand and solve this social problem.

Finally, it would be of value to clarify precisely the nature and targets of perceived prejudice in the judgements our participants currently made. As we suggested above, opponents’ judgements of the relative prejudice of supporting statements may have been made with reference to themselves as the targets of this prejudice. Future work can focus the wording of the items used to measure specifically prejudice towards same-sex attracted people or, say, prejudice towards religious people.

### Conclusion

This research has demonstrated how opposition to same-sex marriage is seen by those holding this oppositional view as tolerant, rational, normative, and even in pursuit of individual liberty *despite* such opposition denying rights to some that are afforded to others. In fact, participants on each side of this debate saw their own perspective as unprejudiced. Recognition of these patterns is important both for theoretical and practical reasons. Theoretically, the data (re)illuminate the traditional “enlightenment” approach to prejudice reduction, reframing supposed educational efforts more as simply social influence. In practice, given influence’s basis in shared group membership [31], what is needed is not pejorative name-calling of opponents as “prejudiced,” but active prejudice-reduction efforts between opponents and supporters to build a common identity [e.g., 71, 72]. As has been detailed elsewhere, this common identity provides a basis for shared values [17], common understandings of fairness and justice [58], and ultimately prejudice reduction [14]. It should also form the basis within which to ground a shared epistemology that will facilitate consensus over facts, and allow for a common recognition of what are and are not prejudiced attitudes. Same-sex marriage supporters have a critical role in this process, as the common identity and shared epistemology could lead to an understanding that actually denies same-sex marriage. Again, we recognize that this is no small effort. But simply labelling opponents as “prejudiced” will most likely entrench the current intergroup divide even further.
